# Where are the female science professors? A personal perspective

**DOI:** 10.12688/f1000research.8889.2

**Published:** 2016-07-14

**Authors:** Shina Caroline Lynn Kamerlin

**Affiliations:** 1 Department of Cell and Molecular Biology, Uppsala University, Uppsala, S-751, Sweden

**Keywords:** Women in science, Implicit bias, Academic gender inequality, Matilda effect, Empowering female academics

## Abstract

The first woman to earn a Professorship at a University in Europe was Laura Maria Caterina Bassi, who earned a professorship in physics at the University of Bologna in 1732. Almost 300 years and three waves of feminism later, in 2016, women typically still only comprise 20% (or less) of the number of full professors in Europe. This opinion article will discuss the experiences of being a female academic today and the factors contributing to the academic gender gap from the perspective of a “young” natural scientist, as well as providing constructive suggestions for strategies to empower women in the academic world.

## Introduction

As women occupy an increasing number of prominent roles in society, it is easy for us to forget just how recent many advances in women’s rights that we currently take for granted actually are. For example, women have only held the right to vote for about 100 years or less in most European countries ^1^. Women have only been allowed to attend European Universities (and initially often only as auditors) since the late 1800s ^2^. In Sweden, before 1859, women did not even have the right to be college teachers ^3^. In such an environment, it is perhaps unsurprising that, when Lise Meitner headed to Germany with the hope of working at the University of Berlin in the early 1900s, women were not even allowed on the premises ^4^. Almost half a century later, when Rosalind Franklin was a research fellow at King’s College London, women were still barred from even entering the senior common room ^5^, effectively cutting them off from college life.

Despite such hostile-to-women environments, female pioneers in science and technology have made countless contributions to science, including developing the first published computer algorithm (Ada Lovelace Byron) ^6^, developing the technique of X-ray crystallography (Dorothy Hodgkins) ^7^, taking the first X-ray diffraction image of DNA (Rosalind Franklin) ^5^, giving us unprecedented insight into the world of chimpanzees, which also helped us better understand ourselves (Jane Goodall) ^8^, co-discovering nuclear fission (Lise Meitner) ^4^ and co-inventing a frequency hopping communication system that is the basis for modern day WiFi technology (Hedy Lamarr) ^9^, to name just a few examples. However, even with the seminal contributions of such women to natural sciences, technology, social sciences and the humanities, we are very far from achieving anything near gender equity at the senior levels of academia. I will explore herein the causes of this inequality and some of the barriers facing women’s progression in the academic world. I will also discuss briefly some of the work I have personally engaged in to fight this inequality, as well as providing suggestions for how we can all contribute to create a more equal working environment.

The focus of this opinion article will primarily be on natural sciences and the academic ladder in Sweden, simply because these are the worlds I know best. I strongly emphasize that this is in *no way* meant to be a value judgment on the Swedish system with respect to other European academic systems, but rather I use Sweden as my example only based on familiarity. Additionally, clearly gender is not the only form of inequality in the academic world, and arguably many other forms of inequality are even more aggravated. The fact that I do not specifically discuss these here is not due to lack of interest, but rather due to the finite scope of this article. Despite these limitations, I believe that many of the discipline- and country-specific challenges we face have universal underlying roots, and therefore that my overall observations are independent of discipline and country. 

## Examining the current state of gender (in)equality in academia

**Figure 1.**
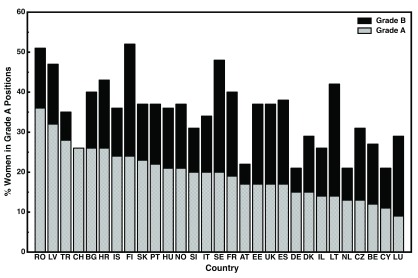
Proportion of female academic staff in Grade A and B positions in Europe in 2010 (for definitions of grades see main text). Data obtained from the 2012 European Commission She figures for gender in research and innovation ^11^. For description of the source data see ref. 11.

In order to set the scene, I would like to start by discussing some statistics of the participation of women in Swedish academia. By all measures, Sweden is a pioneering country in terms of gender equality parameters. For example, Sweden consistently ranks very highly on the World Economic Forum’s Global Gender Gap report, coming in at 4 ^th^ place in 2015, behind only three other Nordic countries (Norway, Finland and Iceland) ^10^. Having achieved such a standing in the Global Gender Gap rankings is something I believe the Nordic countries should justifiably be very proud of. However, despite overall equality in these societies, once one moves to the Academic world, the situation changes rapidly. That is, as can be seen from the European Commission’s 2012 She figures for the % of women in Grade A and B positions in Europe in 2012 ( Figure 1 ^11^), not only does Sweden no longer occupy the top positions for female participation in Grade A positions, it barely makes the European Union average, coming in at 13 ^th^ place. I will return to discuss these statistics later in this opinion article; however, it is worth noting that this comparison also throws up an unexpected surprise: arguably among the most socially conservative countries in Europe, Turkey also has the among the largest number of female professors among European countries (ranking first in 2005 in data by ref. 12), whereas countries such as Germany, Austria, the Netherlands and Denmark, in fact all come in at the bottom half of the list. Note here that a Grade A position is defined by the Commission as the single highest post at which research is normally conducted (in this case a Professorial position), and a Grade B position comprises researchers that are more qualified than newly minted PhD holders, but not as senior as those in Grade A.

**Figure 2.**
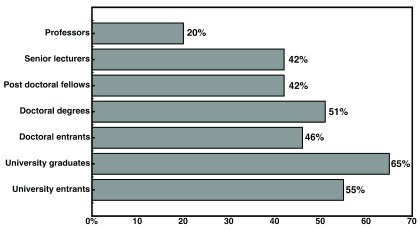
Vertical gender balance in Swedish academia. Data obtained from ref. 14.

**Figure 3.**
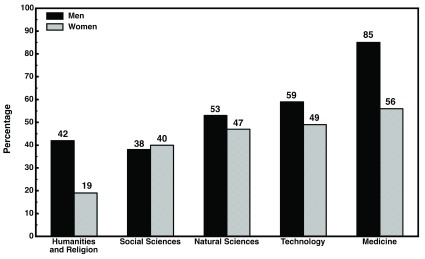
Academic exit rates (in percentages) for men versus women sorted by discipline, measured five years after completion of PhD. Data obtained from ref. 14, based on the 1993 cohort of doctoral students.

There are also a number of stereotypes about women’s involvement in academia that I believe need to be readdressed. For example, when discussions are held about how gender parity could be achieved in academia, it is often asked “why are women leaving?” ^13^. Therefore, I would like to present two more sets of statistics, shown in Figure 2 and Figure 3. Figure 2 shows the percentage of women in different academic career stages after having started a PhD, data from 2013 and averaged over all disciplines ^14^. From this figure, it can be seen that like many other countries, Sweden has a vertical gender balance in academia, such that women comprise the majority (55%) of university entrants, just slightly under the majority of doctoral and postdoctoral candidates, and even senior lecturers/associate professors (46, 42 and 42% respectively), and yet there is a very sharp decline such that this number drops to only 20% of full Professors. This occurs across all disciplines ( Table 1) ^14^, and worryingly even in otherwise heavily female dominated fields such as pharmacology and veterinary medicine. However, importantly, the statistics show that at both undergraduate and doctoral levels, irrespective of the percentages of entrants to these degree programs, women are slightly more likely to stick it out and actually be awarded a degree.

**Table 1.** Percentages of female professors and senior lecturers, as well as total percentage of women in different disciplines in Sweden, based on data presented in 14.

Academic Field	% Female Professors	% Female Senior Lecturers	Total % Women
**Ontology**	24	64	58
**Veterinary medicine**	30	74	71
**Humanities and** **theology**	33	50	49
**Social science**	25	47	47
**Medicine**	24	51	49
**Agricultural science** **and forestry**	23	37	39
**Law**	30	46	44
**Pharmacy and** **pharmacology**	15	65	44
**Natural sciences**	18	27	32
**Mathematics**	11	24	25
**Engineering and** **Technology**	11	24	22
**Total**	**23**	**44**	**43**

Finally, as shown in Figure 3, if one compares the percentage of women and the number of men who have left academia five years after the award of a doctoral degree ^14^, one sees that in all disciplines except social sciences, contrary to stereotypes, more *men* are likely to leave academia than women, although this is then not reflected at the highest levels, for example in the number of full professors. This then raises some really crucial questions: *where did all the women go?* What barriers are facing women in academia today? And how can we empower more women to lead and excel in the academic world?

### Examples of obstacles facing the empowerment of women in academia

Gender studies is a broad field, and many hypotheses have been put forward to rationalize the lack of women in the academic world. Ceci and Williams have summarized these ^15^, providing three general broad arguments that are put forward to explain the dearth of women in academia:

1. The fraction of women who have the native intellectual capacity to do science, particularly at the highest levels, is much smaller than the fraction of men. I should note that I personally find this argument deeply offensive, but it is lamentably a not uncommonly held belief, as was demonstrated in its most high profile example in 2005, when then Harvard President Larry Summers claimed at a conference that the barriers to women’s advancement in academia have been removed, and that the underrepresentation of women at elite universities may stem from “innate” biological differences in ability between the genders ^16^.
2. An inherent lack of interest among women in the hard sciences and engineering.
3. Societal and cultural biases that push women out of the pipeline and lead to the devaluation of those that remain.

I would like to note that Ceci & Williams are not scholars in the field of gender studies. However, the arguments they summarize above align fairly well with my own “on-the-ground” experiences of talking to colleagues in different countries. I will therefore proceed to systematically discuss the main barriers I observe through both my own experiences as a female academic and from discussion with my colleagues below.

### Ongoing challenges balancing career and family obligations for female academics

**

In most European countries, there have been major advances and improvements in mechanisms to allow women to balance career and family obligations. Paid maternity leave, extensions on grants that take into account childcare responsibilities, availability of time off to care for children when they are sick are among only a few of these advances. However, the problem still remains that the crucial formative early years that determine a young scientist’s future career trajectory also coincide in age with the years in which many young scientists need to start seriously considering their family plans. Clearly, balancing the two is not easy, particularly in an environment where hyper-competition is now the norm to attain coveted grants and permanent faculty positions ^17^. Gender studies of course take this challenge into account, and it is a large research area, where the literature can take on scathing titles such as “ *Career progress relative to opportunity: How many papers is a baby ‘worth’?*” ^18^, “ *How much do children really cost?*” ^19^; “ *Balancing work-family life in academia: The power of time*” ^20^, and “ *Pinstripes and breast-pumps: Navigating the tenure-motherhood track*” ^21^. This issue has also been taken up at great length in recent years in editorials and opinion pieces in leading newspapers and magazines, such as Slate Magazine ^22^, the New York Times ^23^, the Chronicle of Higher Education ^24^, and the Atlantic ^25^.

**Figure 4.**
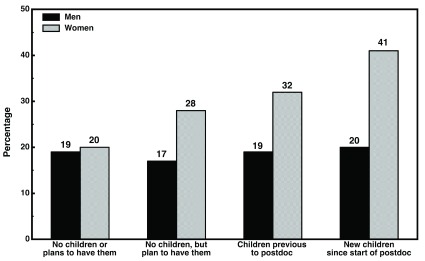
Percentage of postdoctoral researchers who decided against academic research careers, sorted by gender. Results from a 2009 survey of postdoctoral fellows at the University of California, based on data presented in ref. 26.

If one were to summarize the viewpoint of these latter publications on balancing children and an academic career track for female academics, they could be distilled into one simple word: “Don’t”. Additional arguments put forward include that while becoming a parent is not necessarily as bad for a man, having children is a career killer for a woman. This is in practice not always the case, of course, and while children clearly pose a particular challenge, many women have gone on to have successful academic careers while being mothers. Such women, in turn, can make a major contribution as role models for younger colleagues. Additionally, as I will discuss further in this section, ongoing biases against women in academia in my opinion pose a much larger problem than balancing family issues, at least in Sweden. However, it does remain a challenge, as childbirth is often a point at which many women either decide or are forced to leave an academic career trajectory, due to competing personal and professional obligations. For example, a 2009 survey of University of California postdoctoral fellows ( Figure 4) showed that those who already had or were considering having children were more likely to also consider leaving research ^26^. Additionally, the penalties on women who decide to try to have children and not leave research are quite severe. Well-document coping strategies include: waiting until tenure to have children, not having children at all, timing children around the academic calendar, moving to part-time work, increasing research collaborations (presumably to hide “lost time” due to childcare responsibilities), sleeping less, sacrificing personal lives, and moving to “second-tier” institutions ^18^. Clearly, although these strategies are employed as a means to cope, they will have a highly detrimental effect on a female academic’s scientific productivity and career progress.

Additionally, while there has been massive progress in provisions for helping women balance careers and family life, in particular in terms of maternity leave, these are a mixed blessing. Central European countries such as Sweden, Denmark, the Netherlands and Germany have amongst the longest paid parental leave provisions in the world, at 480 ^27^, 364 ^28^, 112 ^29^ and 98 ^30^ days parental leave respectively. One would assume from this that these countries also therefore provide excellent opportunities for women to integrate into the workplace, yet as shown in Figure 1, in particular Denmark, the Netherlands and Germany are among Europe’s poorest performing countries in terms of female integration into the academic world at senior (Grade A) positions. Additionally, Turkey, with the same length maternity leave as the Netherlands (112 days) ^31^, also has among the highest percentage of female professors among European countries.

Clearly, therefore, the link between length of maternity leave and professional success in academia is not as straightforward, and other factors including childcare provisions and societal attitudes play a major role. For example, of the 480 days parental leave in Sweden, 60 days are reserved explicitly for the father, and parents are strongly encouraged to split time equally between them. This would of course have a benefit of distributing the burden of childcare, although there have also been criticisms of the fact that “time” is not divided equally among the genders and the more stressful of childcare duties such as putting uncooperative children to sleep,getting up in the middle of the night to tend to their needs, and the frantic rush to get them out the door in the morning, is more often than not taken on by the female member of the household ^32^. Additionally, even with encouragement to try to split time in Sweden, according to 2012 statistics, women still took 76% of parental leave days with men only taking 24%, and only 13% of parents share parental leave days equally ^33^. Therefore, Sweden is long from splitting parental responsibilities equally between both parents ^34^, and this inequality has been directly linked to both an increased gender-based wage gap and also to hardening the glass ceiling for women in Sweden ^35^. That is, ironically, despite the numerous measures to promote gender equality in the Nordic countries, women in the Nordic countries are actually *less* likely to reach top leadership positions, compared to, for example, the United States, which has fairly minimal regulations with respect to childcare and maternity leave ^36^. This is due to a combination of many factors: actually taking the extended maternity leave options offered can lead to women becoming ect to childcare and e simultaneously becoming rusty on important career skills and social contacts ^36^, which impairs opportunities for further career development. Therefore, while work-family commitments are not the only barrier to the empowerment of women in the academic world, even in 2016, they clearly form a major part of the problem. It is also important to point out however, that as discussed below, implicit biases and structural inadequacies play a very significant role in placing barriers to women’s career progression in academia. While enabling and supporting motherhood is clearly important, there remains a real risk that working out of the “motherhood question” actively obscures other aspects of discrimination, which remain very potent too, and therefore this balance needs to be approached with care.

### Taking on the “Matilda” effect: implicit bias impeding female career progression

In 1968, Merton coined the term the “Matthew” effect, to describe over-recognition of those at the top of the scientific elite, which can extend to even credit misallocation to already well-known scientists ^37^. Following from this, in 1993, Rossiter borrowed this concept to coin the term “Matilda” effect ^38^, which refers to the systematic *under*-recognition of the contributions of female scientists. The question is, therefore, whether such a “Matilda effect” actually exists in science. While I would really like to be able to say no, unfortunately, there is a large amount of qualitative and quantitative evidence pointing to the contrary.

The biggest challenge with the Matilda effect, *i.e.* systematic bias and discrimination against the contributions of women, is that its roots start at a very early age. For example, in 2007, Steinke and coworkers performed the “Draw-a-Scientist” test ^39^. This was essentially a sociological experiment, to get 304 seventh-grade students, to draw what they think a scientist should look like. A summary of the characteristics attributed to male *vs*. female scientists are summarized in Table 2. From the statistics it can be seen that already in the seventh grade, children are heavily influenced by media stereotypes, with the vast majority of children believing that a scientist is a man, in a lab coat and glasses, and 42.4% also assumed that scientists are stern and do not smile. While this may seem whimsical in itself, the implications are severe, because it suggests that already at a young age, children have a distorted image of what it means to be a “scientist” – and therefore a distorted image of their own ability to be an excellent scientist.

**Table 2.** Percentage of Draw-a-Scientist test stereotypes of scientists by gender. Based on data presented in 39.

Question		Girls	Boys	Total
1	Male gender	20.7	36.2	56.9
2	Lab coat	37.5	29.3	66.8
3	Glasses	28.0	29.6	57.6
4	Facial hair	0.7	5.3	5.9
5	Elderly	3.3	8.2	11.5
6	Lab work	17.4	14.5	31.9
7	Work site/laboratory	14.5	13.8	28.3
8	Expression/not smiling	20.4	22.0	42.4
9	Crazy hair	14.8	19.7	34.5
10	Research symbols	14.8	13.5	28.3
11	Knowledge symbols	8.6	10.2	18.8
12	Technology present	0.7	0.7	1.3
13	Indications of danger	0.3	0.7	1.0
14	Signs of secrecy	0.3	0.0	0.3

Unfortunately, the bias that was already being observed in these young middle-schoolers does not go away, but rather is consolidated as the children grow up progress through the academic ranks. For example, in 2010, Amy Bug from Swarthmore College performed another sociological experiment, in which 126 students had to watch 4 ten-minute lectures given by two male and two female physics professors ^40^. The students then had to evaluate both the lecture, and the professor’s knowledge ability. What Bug observed was that, on average, female students gave slightly higher marks to the women than to the men, but that this was more than compensated for in the fact that male students on average gave *massively* higher marks to men than to women. In addition, neither group was aware of the fact that their professors were paid actors, reading from exactly the same script, with no prior background in physics!

**Figure 5.**
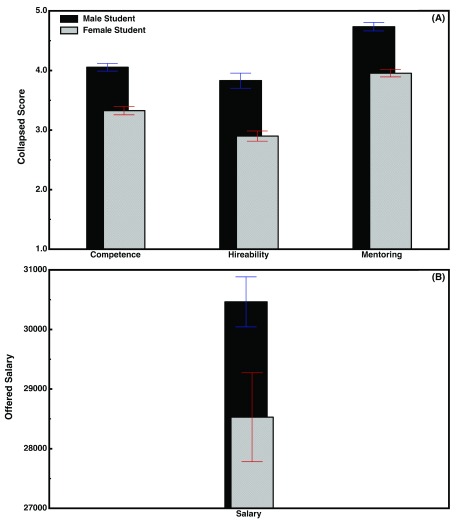
Collapsed results, independent of gender of evaluator, showing ( **A**) competence, hirability and mentoring scores (assessed on a scale from 1 to 7), and ( **B**) offered salaries, for male and female students. Based on data presented in ref. 41. Error bars represent standard deviation over the two genders. For methodological details and raw data, see ref. 41.

Taking this one step further, when it comes to recruitment of undergraduate lab assistants, Moss-Racusin and coworkers ^41^ performed a randomized double blind study in which a broad, US-wide sample of science faculties ( *n*=127) received a hypothetical application pack for recruitment to a position as an undergraduate laboratory assistant. The materials were randomly assigned either a male ( *n*=63) or female ( *n*=64) name. All other parameters were *identical*. Faculty members were then asked to rate students’ competence, hirability, and the salary and mentoring they would offer the student. The results of this are shown in Figure 5. Critically, all faculties believed that students would see the feedback. From this figure, it can be seen that not only were “male” lab assistants routinely deemed to be more hirable, competent and worthy of mentoring, the salary gap for applicants with *exactly the same* CV was in excess of $3000/year. Additionally, *both* male and female faculty members judged the female student as less competent, and less worthy of being hired than an identical male applicant, and also offered her less salary and mentorship. This faculty member bias was observed to be independent of gender, scientific discipline, age and tenure status. Female and male faculty members are equally biased. While this may in itself again seem to be just a trivialized local study, clearly such subconscious bias can in turn translate into large real world disadvantages in the judgment and treatment of female students. This in turn raises concern about the extent to which negative pre-doctoral experiences may shape women’s subsequent career decisions ^41^.

Finally, in a by now quite famous study on sexism in Swedish peer review ^42^, the authors explored the discrepancy between the fact that in 1997, women were awarded 44% of Swedish biomedical PhDs, but held only 25% of postdoctoral and 7% of professorial positions (a statistic that Figure 1 shows has fortunately almost tripled in under 20 years). Additionally, the success rates of women applying to prestigious medical research council (MRC) postdoctoral fellowships was only half that of male applicants. The reasons put forward to justify this were variants on the theme presented at the start of this section, that women are “less productive”, “less motivated”, “less career oriented”, “less …”. Unconvinced, the authors decided to explore whether reviewers can truly do a “gender-free” evaluation. At the time, the postdoctoral fellowship applications comprised of a *Curriculum Vitae*, publications list and proposal. These were then reviewed by five people from one of 11 topical evaluation committees. Each reviewer awarded the applicant a score of between 0 and 4 each for scientific competence, relevance of the research proposal, and the quality of the methodology. The scores were then multiplied to give a product score between 0 and 64, and averaged over all five reviewers to give a final score to the applicants, with candidates being ranked according to the final score received. Under Sweden’s Freedom of the Press Act, the authors were allowed to see the MRC evaluations by court order. From this, they observed that female applicants had lower scores on all three evaluation points, and that these were, on average, 0.25 lower for competence, 0.17 lower for methodology and 0.13 lower for the quality of the research proposal. The multiplicative nature of the different criteria then led to substantially lower overall final scores.

What stood out from this assessment was the fact that the female candidates appeared to be deemed particularly deficient in scientific competence compared to their male counterparts. Since assessment of scientific competence is normally related to the number and quality of the applicant’s scientific publications, the authors wondered if the female applicants are really less productive than the male ones. To assess this, the authors constructed a model, in which each applicant was given a “mean competence score”, as a function of scientific productivity, measured as total impact. On top of this, the authors used multiple regression analysis to correct for external factors such as nepotism, university affiliation, and connections to members of the evaluation committee. Once completed, the authors observed that, even after correction, the female applicants needed to, on average, be *2.5 times as productive* as man for the same competence score, with a worrying trend that the higher impact the applicant, the higher a male applicant’s contributions were scored compared to their female counterpart (an extreme incarnation of the Matilda effect). In concrete terms, this translates to three extra papers in Nature or Science, or 20 extra paper in a journal with an impact factor of 3, and with such expectations it would hardly be surprising that fewer women manage to achieve academic career success than men.

Clearly, the Swedish Research Council responded strongly to these observations, and put systems in place to improve the peer review process and promote applicants receiving equal treatment irrespectively of gender. While things have improved dramatically since then, in particular with women receiving 35% of grants awarded by the Swedish Research Council for 2015 ^43^, there are still clear areas that need addressing before a truly “gender free” peer review process can be achieved ^44^. Additionally, clearly gender bias in peer review is far from a uniquely Swedish problem, and a 2007 meta-analysis of 21 such studies demonstrated that, on average, male applicants have a 7% greater change of obtaining research funding than female applicants, which can be quite a dramatic difference at a time when grant success rates are going into the single digits ^45^. This is problematic, in particular in light of the fact that in such a low-success environment, even small biases can have major negative impact ^46^ (creating a “mountain of feathers”). Many other examples of the Matilda effect have also been observed, in selecting women for conference presentations ^47, 48^, assessing publication quality and citations ^49, 50^, recruitment and tenure processes ^51, 52^, and even in recent arguments that elite male faculty members in the life sciences employ fewer women than men to their labs, thus creating an unbalanced career start for young female scientists ^53^, or that papers in which the lead author is a man are more likely to get cited than corresponding work led by a woman ^49^. Therefore, unfortunately, the Matilda effect is alive and well in science, and one of the biggest current barriers to the true empowerment of women in the academic world.

## Focus 2016: how can we empower women in the academic world?

Having explored some of the major barriers to women in the academic ladder, I would like to focus on constructive examples of how these barriers can be removed, in order to achieve greater gender equity in academia.

### The Turkey example: how did Turkey beat these unfavorable odds?

**

To open this section, I would like to briefly come back to the example of Turkey, which is leading in Europe in percentage of women in academic positions ^12^. In some cases where women are highly represented in senior academic positions ( Figure 1), it has been argued that this is in part because of the willingness of women to take more insecure and poorly paid career trajectories, and that academia in general is considered a less prestigious career path, thus increasing female representation in this sector (see for example ref. 54, and references cited therein). However, in this respect, Turkey’s example is therefore worth highlighting, because the high female representation in Turkey is not by accident, but rather a result of concrete policies over a longer period of time. In particular, Healey and coworkers argue that the higher representation of Turkish women in academia can be brought down to five key features of the Turkish system ^55^:

1. The existence of historical long-term state-driven ideology, promoting the participation of women in the Turkish academic labor force.
2. The fact that, in general, academia has been considered a “female appropriate” career choice, resulting in little gender disparity among university graduates (even in the sciences).
3. The existence of significant university expansion in the 1990s, which created demand for both male and female professors.
4. The existence of a comparatively transparent employment and promotion system.
5. The reliance of female faculty on domestic help, making it easier to balance family and professional commitments.

Clearly, when these factors are brought together, they lead to a holistic picture that is productive for the empowerment of women in the academic world, and show that even though there are many structural and practical problems that still need addressing, nevertheless, the barriers facing the empowerment of women in the academic world are surmountable ones, if we are only willing to take them on. I would like to note, however, that every silver lining has a cloud, and as pointed out by a reviewer of this opinion peace, that while career-promoting, the reliance on domestic help among academic professors simply redistributes domestic labor among (mainly) women and other categories of class and ethnicity.

### Mentorship and raising the visibility of academic women

A few years ago, in discussion with a postdoctoral scholar in a colleague’s research group about her career prospects, she asked me why she should even remain in academia, when there are no women. This remained with me and is in part the reason for why I have taken a lot of the mentorship work I discuss in the concluding section. Young women need strong role models: it is important for successful women in academia to be one. This can be achieved in many ways. Mentoring of junior colleagues is particularly important, as is encouraging them to actually apply for grants, fellowships, faculty positions and promotions. In my work mentoring junior colleagues, I often hear my mentees insist that they are not yet ready to do so, what if they only had just a few more papers, the call for appointment is not really in their field, and maybe they can apply the next year. This creates a problem, because it means that many women don’t think they are good enough, and don’t get on the academic ladder in the first place. This can be addressed through greater mentorship opportunities, as well as raising the visibility of women who do exist in academia.

To partly address this issue, I have together with the Young Academy of Europe and Uppsala SciLifeLab organized a one-day symposium at Uppsala University, with 12 outstanding speakers from disciplines across natural sciences and technology, and four similarly prestigious session chairs ^56^. This was tremendously successful, with the participation of 166 delegates from 12 different countries and four continents. Additionally, the University of Southern California Women in Science and Engineering (WiSE) program have compiled a database of women in theoretical/computational chemistry, material science and biochemistry ^57^. In these fields, women provide only a smaller percentage of total faculty in any given department, and can therefore easily be lost in the crowd at individual institutions. However, this database highlights the fact that globally, there are several hundred examples of women working in these research areas, and provides for example a quick reference list of outstanding women one can refer to when putting together seminar series, conference speaker lists, and similar activities. Such lists can provide a quick reference point for conference organizers who want to ensure a more equal gender distribution when planning meetings and symposia, by highlighting outstanding women in different research areas. This is particularly important in light of the ongoing poor gender distribution among invited speakers for many key conferences, as was for instance highlight in the recent controversy with regard to the speakers list for the 15 ^th^ International Congress in Quantum Chemistry (ICQC), which is the triannual flagship conference of the International Academy of Quantum Molecular Science ^48, 58^, to name just one example.

Finally, in addition to giving (academically) younger women more confidence, how we represent and promote our junior colleagues is also critical. On this note, there has been an interesting study examining the gendered aspects of letters of recommendation written at a large American medical school in the mid-1990s, and clear differences were observed in letters written for male and female applicants ^59^. This included not just lengths of the letters and the kind of language used, but also gendered representations in the letters themselves. This ties in, to some extent, also with the issue of implicit bias, and something we need to be aware of when promoting our junior colleagues, and assessing those who have trusted us with recommending them for their future careers.

### Raising awareness of implicit bias

As discussed in this contribution, a major contribution to the low percentages of female science professors is the existence of a “Matilda effect” in science, that manifests itself from a very early career stage, and which women fall as easily prey to the exercising of as men do. Here, there have been significant advances in strategies to address implicit bias in the workplace, as well as in funding and promotion panels and peer review (a quick internet search on this topic will provide countless hits), and I would also strongly recommend taking an implicit association test such as that provided by Harvard University ( https://implicit.harvard.edu/implicit/takeatest.html) to test your own implicit biases. Unfortunately, by the very nature of being “implicit” we all carry some level of bias, and self-awareness and self-correcting for our biases can go a very long way towards fighting the Matilda effect in science. Finally, it is clear that implicit bias is not the only barrier facing women in academia, and thus awareness of this issue will not somehow magically make all other problems go away. Nevertheless, it is impossible to fix a problem one doesn’t know exists, and therefore I personally believe that implicit bias training should be an important pre-requisite of preparation of decision makers before serving for example on grant award and recruitment committees. In this way, although not foolproof, candidates would nevertheless have an elevated chance of being judged primarily on merit.

## Conclusion

In this opinion article, I have discussed at length the role in which explicit and implicit bias, both in terms of external perceptions and personal perceptions of one’s competence and ability, can play as barriers to female progression in academia. As a tenured faculty member working in computational biology (which is a research area which still maintains lower participation of women), I put my academic success strongly down to the fact that from an early stage, I had very strong role models giving me support and encouragement, and believing in my ability to achieve this. I believe, therefore, it is extremely important to give back to other younger colleagues, to give them the same opportunities and support to succeed in a system where the odds are still stacked against female academics. To facilitate this, I actively recruit and mentor highly promising young women to my research team, and take great pleasure from watching their own career success in turn. Here, I do my best to pay particular care to the knowledge that in the Matilda effect, women are just as biased as men. Amelia Earhart once said, “ *Women must try to do things as men have tried. When they fail, their failure must but be a challenge to others*” ^60^. Tremendous contributions have been made by structured programs to increase the presentation of woman in senior academic positions, such as the NSF Advance program ^61^ in the US, or the Athena Swan program in the UK ^62^. Ultimately, however, academia is comprised of each and every one of us, and it is the choices we make that will determine the future representation of women in the academic world.
