# Supplementary material for: Where are the female science professors? A personal perspective
Source: F1000Res. 2016 Jul 14;5:1224. Originally published 2016 Jun 6. [Version 2] doi: 10.12688/f1000research.8889.2 (PMC4909113; doi:10.12688/f1000research.8889.2)
Supplement: Peer review history

# Peer review: Referee response for version 2

**DOI:** 10.5256/f1000research.9927.r14999

**Published:** 2016-07-14

**Authors:** Pernilla Wittung-Stafshede

**Affiliations:** 1 Department of Biology and Biological Engineering, Chalmers University of Technology, Gothenburg, Sweden

I have read this submission. I believe that I have an appropriate level of expertise to confirm that it is of an acceptable scientific standard.

# Peer review: Referee response for version 1

**DOI:** 10.5256/f1000research.9566.r14198

**Published:** 2016-06-24

**Authors:** Ruth Müller

**Affiliations:** 1 Munich Center for Technology in Society, Technical University of Munich, Munich, Germany

I enjoyed reading this opinion piece. The author, a researcher from the natural sciences, engages with different quantitative and qualitative studies from gender studies and science & technology studies and relates the results on how gender influences career development and recognition to her own experiences. She clearly states that this is the position she is writing from and hence adequately situates her contribution. 

 I have minor comments to improve this opinion piece:

 First, I am not sure what purpose the section that discusses Ceci & William's work serves within the opinion piece; the section seems disconnected from the rest of the piece; I think this section could be left out or rewritten. For a rewrite I want to note that Ceci & Williams are not scholars in the field of gender studies, but rather study sex differences (e.g. in relation to I.Q. or career choice).

 Second,  the author draws our attention very clearly to how gender discrimination is not only a question of parenthood but based on deeply rooted stereotypical assumptions about the interests and capabilities of the dichotomously constructed categories of women and men. One could add that reframing gender discrimination as mainly a problem of working out the "motherhood question" and enabling and supporting motherhood actively obscures other aspects of discrimination, which, as the author elaborates, are very potent factor, too.

 Third, I would like to draw attention to how in the Turkey example the reliance of academic professors on domestic help may be very favorable for their careers, at the same this constellation redistributes domestic labour mainly among women and along categories of class and ethnicity. I would invite the author to mention that.

 Forth, with regard to the "Raising awareness of implicit bias" section, I would invite the author to discuss the question if decision makers (referees for grants, hiring committees... etc) should undergo gender bias awareness training before being granted the power to decide? This has been a controversial question and I am interested in the authors opinions.

 Last, as the author, much to my pleasure, is interested in concrete action to better the situation, I would like to draw attention to a study by Trix & Psenka from 2003 that describes gender bias in recommendation letters. I find this study instructive for checking you own language in describing and assessing those who trust you to recommend them for career relevant positions. http://das.sagepub.com/content/14/2/191.abstract

I have read this submission. I believe that I have an appropriate level of expertise to confirm that it is of an acceptable scientific standard.

## References

[1] TRIXFPSENKAC: Exploring the Color of Glass: Letters of Recommendation for Female and Male Medical Faculty. *Discourse & Society*.2003;14(2) : 10.1177/0957926503014002277 191-220 10.1177/0957926503014002277

# Response

**Published:** 2016-07-10

**Authors:** Lynn Kamerlin

**Affiliations:** Department of Cell and Molecular Biology, Uppsala University, Sweden

I am delighted that the reviewer enjoyed reading the opinion piece, and appreciative of the constructive suggestions for further improving it. Based on the reviewer’s suggestions, a revision has now been submitted in which the following modifications have been made to the manuscript:

  

- With regard to the work of Ceci & Williams, although the authors are not scholars in the field of gender studies, the views they summarize align fairly well with my “on the ground” experiences. This has now been stated explicitly in the manuscript to explain what that section is doing there.

 

- A statement has been added cautioning against allowing the “motherhood question”, which remains a major challenge, to take over and obfuscate other aspects of discrimination, which are also very potent, and thus why it’s important to approach this issue with care.

 

- A statement has been made about the problems with an over-reliance on domestic help simply redistributing the problem amongst women and other categories of class and ethnicity.

 

- A statement has been made about implicit bias training for decision makers before sitting on grant and hiring committees. Although this is controversial and only raises awareness of rather than solves problems, nevertheless it’s an important first step. Here, I would like to also point out that implicit bias is not only a gender issue, but involves also bias based on ethnicity, academic rank, geographic location, and any other number of variables, and poses a significant problem in grant evaluation and recruitment processes. Therefore, implicit bias training should not be limited to gender awareness alone.

 

- A short paragraph discussing gender bias in recommendation letters (based on the study of Trix and Penska from 2003) has been now included in the manuscript.

# Peer review: Referee response for version 1

**DOI:** 10.5256/f1000research.9566.r14201

**Published:** 2016-06-20

**Authors:** Sarah de Rijcke

**Affiliations:** 1 Centre for Science and Technology Studies, Leiden University, Leiden, Netherlands

This is an excellent, well-written and well-researched opinion paper about barriers to female progression on the academic career ladder. Though Kamerlin carefully positions herself in the context of the Swedish research system and in the natural sciences, her observations (unfortunately) apply to many more contexts and fields.

 I would like to pick up on one point that Kamerlin discusses at quite some length: the issue of implicit bias. This is an issue facing many women from very early on in their academic career, as a result of deeply entrenched stereotypes about what constitutes a proper scientist, what a scientist looks like and what a scientist does. Kamerlin rightly points out how these implicit biases affect women's own career choices, in addition to the hiring and promotion procedures they are subjected to, and even the peer review of their grant proposals and papers.

 There is one point about implicit bias that the author leaves untouched though in an otherwise broad-ranging and very comprehensive article. And this is the point about how *academic merit itself is *not objective and a-political, but *heavily masculinised*. I recommend reading the excellent article by Margaret Thornton on the contemporary re-masculinisation of the academy (Thornton, 2013), in which she shows how academic capitalism also exercises incidental gender effects. In today's highly competitive and precarious climate, Thornton argues, researchers of any gender are incentivised to focus mainly on masculinised performance measures that promote productivity and accumulation of capital. In the process, other - feminised - activities are seen as unproductive. This includes pastoral care for students, thinking, reading, anonymous reviewing, and mentoring of junior researchers. What counts is that which can be counted. Anything else is rendered as 'waste'.

 I hope that discussions about barriers to female career progression will also spearhead into an open debate about *the kind of science we currently hold in highest regard in the first place*. Does 'the gendered sub-text of technopreneurialism' (Thornton, 2013: 134) override more complex notions of quality and academic virtues? Does 'benchmark masculinity' (ibid: 136) affect the kinds of knowledge we produce under such conditions?

 That said, I wholeheartedly agree with Kamerlin that we need more mentors, and more role models. And she is definitely one of them. Many researchers should find inspiration in how Kamerlin uses the networks and platforms at her disposal to further the discussion about the gender gap in the academy, and how she is putting into practice a different set of leadership skills, in order to make a difference.

I have read this submission. I believe that I have an appropriate level of expertise to confirm that it is of an acceptable scientific standard.

## References

[1] ThorntonM: THE MIRAGE OF MERIT. *Australian Feminist Studies*.2013;28(76) : 10.1080/08164649.2013.789584 127-143 10.1080/08164649.2013.789584

# Response

**Published:** 2016-06-20

**Authors:** Lynn Kamerlin

**Affiliations:** Department of Cell and Molecular Biology, Uppsala University, Sweden

Thank you for your careful reading of the manuscript, and for your kind words, both of which are much appreciated. Thank you also for the valuable reference, which covers a point that is often overlooked, and I think actually very important in terms of understanding the parameters shaping our experiences as researchers. One can only hope that such researcher trickles down to practitioners to cause a paradigm shift towards a more healthy working environment for the next generation of academics.

# Peer review: Referee response for version 1

**DOI:** 10.5256/f1000research.9566.r14351

**Published:** 2016-06-14

**Authors:** Anna I. Krylov

**Affiliations:** 1 Department of Chemistry, University of Southern California, Los Angeles, CA, USA

This is an excellent paper discussing the factors responsible for slow advancement of women in academe. The author points out to societal pressures and expectations, uneven distribution of family responsibilities, and unconscious biases. Her points are well documented and supported by data and numerous studies.

 As far as biases are concerned, I would also mention Steinpreis et. al, Sex Roles, v. 41 (1999). In this study, identical academic resumes (differing only by the gender of the candidate) were mailed for expert evaluation for hireability and tenurability. The outcome was that the male name on the resume resulted in higher competence scores. Particularly disconcerting was that female tenure candidates were four times as likely to receive cautionary comments such as "We would have to see her job talk", "I would need to see evidence that she had gotten these grants and publications on her own", "It is impossible to make such a judgment without teaching evaluations".

 While the existence of biases has been well documented and is now reluctantly recognized in the community, the implications of societal pressures and cultural expectations are usually not openly discussed. I applaud the author for pointing out that what some consider to be family friendly practices, such as unnecessary long maternity leaves practiced in Nordic countries, are hugely detrimental for women's advancement and gender equality. What is needed is not long maternity leaves, but the availability of high-quality affordable child care and domestic help.

 In my opinion, outdated societal expectations and cultural reality are primarily responsible for women dropping out from the workforce. Even when child-care options are available, there is an expectation that mothers have to provide personal day-to-day care to their children, be involved in school activities through volunteering, provide support for extra-curriculum activities, etc. These expectations stem from the culture of  stay-home moms or women choosing mommy-track (which exists both in the US and in Europe) leads to over-parenting practices and creates a pressure for normal women to follow the suit. I believe we cannot achieve true gender equality as long as it is considered to be acceptable to be married to a stay-home wife.

 Besides the child-care practices, broader implications of this is that men in traditional marriages contribute to perpetuation of biases and create an unhealthy workplace environment. This is documented in the following study: Desai, Sreedhari D. and Chugh, Dolly and Brief, Arthur, The Organizational Implications of a Traditional Marriage: Can a Domestic Traditionalist by Night be an Organizational Egalitarian by Day? (March 12, 2012). UNC Kenan-Flagler Research Paper No. 2013-19. The quote below illustrates the point.

 "Based on five studies with a total of 993 married, heterosexual male participants, we found that marriage structure has important implications for attitudes, beliefs, and behaviors related to gender among heterosexual married men in the workplace. Specifically, men in traditional marriage (married to women who are not employed) disfavor women in the workplace and are more likely than the average of all married men to make decisions that prevent the advancement of qualified women. Results show that employed men in traditional marriages tend to (a) view the presence of women in the workplace unfavorably, (b) perceive that organizations with higher numbers of female employees are operating less smoothly, (c) perceive organizations with female leaders as relatively unattractive, and (d) deny qualified female employees opportunities for promotions more frequently than do other married male employees."

 In sum, this is an excellent viewpoint paper. I will recommend it to others as a valuable resource. My only criticism of the paper is the choice of the venue for its publication. The author should have submitted this paper to a reputable journal adhering to established publication and peer-reviewing practices.

I have read this submission. I believe that I have an appropriate level of expertise to confirm that it is of an acceptable scientific standard.

## References

[1] SteinpreisRAndersKRitzkeD: *Sex Roles*.1999;41(7/8) : 10.1023/A:1018839203698 509-528 10.1023/A:1018839203698

[2] DesaiSChughDBriefA: Marriage Structure and Resistance to the Gender Revolution in the Workplace. *SSRN Electronic Journal*.2012; 10.2139/ssrn.2018259 10.2139/ssrn.2018259

# Response

**Published:** 2016-06-14

**Authors:** Lynn Kamerlin

**Affiliations:** Department of Cell and Molecular Biology, Uppsala University, Sweden

Thank you very much for the thoughtful and valuable comments on the paper, and also for the useful additional references. I am very glad that the reviewer appreciated the work, and also agree fully with all the additional points raised in the report. From the two referee reports it's clear that this is an issue that is significantly under-discussed, but that resonates strongly with other female professors as well, and I hope this encourages greater debate around (and constructive solutions to) this issue.

# Peer review: Referee response for version 1

**DOI:** 10.5256/f1000research.9566.r14197

**Published:** 2016-06-07

**Authors:** Pernilla Wittung-Stafshede

**Affiliations:** 1 Department of Biology and Biological Engineering, Chalmers University of Technology, Gothenburg, Sweden

This is an excellent text on gender bias in academia with a focus on Sweden but with a general perspective. The topic is highly important, especially since people in many countries believe Sweden is a gender equal country.

 As the author clearly demonstrates with facts and numbers, this is not true. The text is well written: it contains a historical perspective and the most famous studies demonstrating gender bias in academia are described.

 The solution that senior women must mentor younger women and help them believe in themselves and pursue careers in academia is very good. Nonetheless, I believe more must be done at Swedish universities to achieve a true change in a foreseeable future. All faculty and administrators need to be educated about gender bias, both conscious and unconscious, and every academic leader must strive for equal treatment in every situation and decision. See below where I outlined some actions to take around the same topic:

http://www.stemwomen.net/is-the-gender-gap-solved-in-liberal-sweden/

I have read this submission. I believe that I have an appropriate level of expertise to confirm that it is of an acceptable scientific standard.

# Response

**Published:** 2016-06-07

**Authors:** Lynn Kamerlin

**Affiliations:** Department of Cell and Molecular Biology, Uppsala University, Sweden

I would like to thank the reviewer for the positive assessment of my perspective piece, and also for the link to their related piece in STEM Women. It is clear from that piece that the problems I describe in my work only increase as one moves up the seniority ladder, and therefore it is refreshing to see the constructive 6-point plan at the end of the STEM Women piece. In particular, I fully agree with the importance of gender bias training, since the nature of implicit biases is that they are implicit, and therefore the people who carry them are not even aware that this is the case. I hope that debate about this issue increases, as well as proactive action, so we can move towards true equality of opportunity in academia.
